# Inhibition of FEN1 Increases Arsenic Trioxide-Induced ROS Accumulation and Cell Death: Novel Therapeutic Potential for Triple Negative Breast Cancer

**DOI:** 10.3389/fonc.2020.00425

**Published:** 2020-04-03

**Authors:** Xing Xin, Ti Wen, Li-Bao Gong, Ming-Ming Deng, Ke-Zuo Hou, Lu Xu, Sha Shi, Xiu-Juan Qu, Yun-Peng Liu, Xiao-Fang Che, Yue-E Teng

**Affiliations:** ^1^Department of Medical Oncology, The First Hospital of China Medical University, Shenyang, China; ^2^Key Laboratory of Anticancer Drugs and Biotherapy of Liaoning Province, The First Hospital of China Medical University, Shenyang, China; ^3^Department of Respiratory and Infectious Disease of Geriatrics, The First Hospital of China Medical University, Shenyang, China

**Keywords:** FEN1, ROS, arsenic trioxide, GSH, Nrf2

## Abstract

Triple-negative breast cancer (TNBC) is an aggressive subtype of breast cancer, which is very difficult to treat and commonly develops resistance to chemotherapy. The following study investigated whether the inhibition of Flap Endonuclease 1 (FEN1) expression, the key enzyme in the base excision repair (BER) pathway, could improve the anti-tumor effect of arsenic trioxide (ATO), which is a reactive oxygen species (ROS) inducer. Our data showed that ATO could increase the expression of FEN1, and the knockdown of FEN1 could significantly enhance the sensitivity of TNBC cells to ATO both *in vitro* and *in vivo*. Further mechanism studies revealed that silencing FEN1 in combination with low doses of ATO might increase intracellular ROS and reduce glutathione (GSH) levels, by reducing the nuclear translocation of nuclear factor erythroid 2-related factor 2 (Nrf2); elevating ROS leaded to apoptosis and p38 and JNK pathway activating. In conclusion, our study suggested the combination of FEN1 knockdown and ATO could induce TNBC cell death by promoting ROS production. FEN1 knockdown can effectively decrease the application concentrations of ATO, thus providing a possibility for the treatment of TNBC with ATO.

## Introduction

Breast cancer is a disease with the highest morbidity in malignant tumors in women (1). Based on the expression of estrogen receptor (ER), progesterone receptor (PR), and human epidermal growth factor receptor 2 (HER 2) in tumor cells, breast cancer can be divided into multiple subtypes. TNBC, a subtype accounting for about 20% of all breast cancers (2, 3), tends to be more aggressive and difficult to treat. Especially, the women under the age of 40 years old with TNBC has poor clinical outcomes and disproportionately higher prevalence (4). Nowadays, although chemotherapy is considered the main treatment approach for TNBC patients; followed by surgery and radiotherapy, the treatment effect is limited, and most patients occur disease progression in a relatively short time span (5).

It is well-known that many anti-cancer drugs can cause cellular DNA damage and induce apoptosis by promoting ROS production in tumor cells. However, ROS-induced oxidative DNA damage can be repaired by the BER pathway, which is considered as one of the mechanisms responsible for chemotherapy resistance (6). FEN1, the key protein of the BER pathway, participates the repair of DNA damage by removing the 5′-flaps produced by the Polδ/ε (7, 8). Previous reports have shown that FEN1 is highly expressed in several types of cancer (9–12), and significantly reduces the efficacy of anti-tumor drugs (9, 13). In non-small-cell lung carcinoma (NSCLC), FEN1 inhibition can promote apoptosis of tumor cells, in turn leading to higher sensitivity to cisplatin (9). Many studies have shown that FEN1 is highly expressed in proliferative cancer cells and is essential for cell growth in tumor tissues (14, 15). In addition, recent study has also reported that the inhibition of FEN1 phosphorylation decreased the tolerance of myocardial cells to the high oxygen environment during perinatal period, thus causing oxidative damage (16), which is mainly triggered by ROS accumulation (17). However, it is not clear whether FEN1 deficiency can affect the ROS levels in tumor cells, high expression of FEN1 might effectively repair the oxidative damage, leading to drug resistance in TNBC cells.

ATO has been proved to cause ROS increase in many tumors, including acute myeloid leukemia, hepatocellular carcinoma, and lung cancer (18–20); while its effect on TNBC has not yet been investigated. Therefore, in this study, we aimed to use a ROS inducer ATO in combination with FEN1 inhibition to investigate whether FEN1 is correlated to ROS production or accumulation. On the other side, we attempted to elucidate whether ATO could maintain the anti-tumor efficiency at low doses in TNBC; at high concentration ATO can cause side effects, including hepatotoxicity neurotoxicity, nephrotoxicity, cardiotoxicity, and risk of dermatological diseases, which limits it's application in clinic (21).

We confirmed that targeting FEN1 could enhance the efficiency of ATO to TNBC cells by increasing the level of ROS. This phenomenon was caused by reduced nuclear translocation of Nrf2, which led to a GSH depletion and ROS accumulation. This study provided the theoretical basis for clinical application of ATO to TNBC patients, and revealed the potential of FEN1 as a potential therapeutic target for TNBC.

## Materials and Methods

### Cell Culture and Reagents

Human breast cancer cell lines, MDA-MB-231 and MDA-MB-468, were purchased from the Cell Bank of Type Culture Collection of the Chinese Academy of Sciences (Shanghai, China). All the cells were maintained in Leibovitz's L-15 medium (Gibco) supplemented with 10% fetal bovine serum (Gibco-BRL), and grown under an atmosphere of 5% CO_2_ at 37°C.

### MTT Assay

The 3-(4,5-dimethyl thiazol-2-yl)-2,5-diphenyl tetrazolium bromide (MTT) assay was used to measure the effects of ATO and/or FEN1-knockdown (KD) on cell proliferation. The MDA-MB-231 cells (7 × 10^3^ cells/well) and the MDA-MB-468 cells (6 × 10^3^ cells/well) in 96-well plates, were exposed to various concentrations of ATO with or without FEN1-KD and for the indicated times. Thereafter, 20 μL of MTT solution (5 mg/mL) was added to each well, and the cells were incubated for another 4 h at 37°C. Then the cell culture medium was removed and 200 μL of dimethylsulfoxide (DMSO) was added. The optical density (OD) was measured at 570 nm with a microplate reader (Model 550, Bio-Rad Laboratories, USA).

### Cell Transfection

The MDA-MB-231 and MDA-MB-468 cells at a density of 3 × 10^5^ were transfected using Lipofectamine® 2000 transfection reagent (Invitrogen; Thermo Fisher Scientific, Inc.). The negative control (NC) and FEN1 siRNA sequences from Guangzhou RiboBio Co., Ltd., (Guangzhou, China) were as follows: NC forward, 5′-UUCUCCGAACGUGUCACGUTT-3′ and reverse, 5′-ACGUGACACGUUCGGAGAATT-3′; FEN1 forward, 5′-GGGUCAAGAGGCUGAGUAAdTdT-3′ and reverse, 5′-dTdTCCCAGUUCUCCGACUCAUU-3′. The NC or FEN1 siRNA (10 nM) and Lipofectamine® 2000 were diluted in L-15 medium. Following 20 min of incubation at 37°C, the complexes were added to each well of 6-well plates containing serum-free L-15 and cells. Following 72 h of transfection, cells were used in the subsequent experiments.

### Measurement of ROS Generation

ROS were detected by carboxy-H2DCFDA (C6827, Invitrogen, USA). The MDA -MB-231 and MDA -MB-468 cells were treated with FEN1-KD and/or ATO for 48 h, followed by pretreatment with or without N-acetyl-L-cysteine (NAC, 10 mM) for 30 min. The cells were stained with 1 mM carboxy-H2DCFDA for 30 min at room temperature, then resuspended in phosphate buffer saline (PBS) for analyzing by flow cytometer (Becton-Dickinson, USA).

### Detection of Intracellular GSH

Cellular GSH levels were analyzed using 5-chloromethylfluorescein diacetate (CMFDA, Ex/Em=522/595 nm, Invirtogen, CA, USA) fluorescence. In brief, cells with different treatment were washed with PBS and incubated with 1 μM CMFDA at 37°C for 15 min according to the manufacturer's instructions, followed by the incubation for 40 min at 37°C with complete medium. CMF fluorescence was determined by flow cytometer. For each sample, 5,000 events were collected.

### Immunofluorescence

MDA-MB-231 cells seeded in Lab-Tek chamber slides (Nunc S/A, Polylabo, Strasbourg, France) were treated with different conditions, and fixed in 3.3% paraformaldehyde for 20 min. Then the slides were permeabilized with 0.2% Triton X-100 for 5 min and blocked with 5% bovine serum albumin (BSA). For double staining, they were primed with anti-γ-H2AX mouse antibody (Cell Signaling Technology, MA, USA) overnight at 4°C. Next day, after incubated with Alexa Fluor 568-conjugated goat anti-mouse IgG (Thermo Scientific, MA, USA) in 1% BSA for 1 h at room temperature, cells were further incubated with 4060-diamidino-2-phenylindole (DAPI) for 5 min for nuclei staining. Finally, the slides were mounted and visualized by fluorescence microscopy (BX61, Olympus, Japan).

### Apoptosis Analysis by Flow Cytometry

The apoptosis of MDA -MB-231 and MDA -MB-468 cells with different treatment was detected by using the eBioscience™ Annexin V-FITC Apop Kit (BMS500FI-300, Invitrogen, USA). The collected cells were washed with PBS and resuspended in binding buffer at the density of 3 × 10^5^ cells/mL. Then, the cells were double stained with Annexin V-FITC and propidium lodide (PI). Apoptosis was analyzed by flow cytometry.

### Antitumor Effect on Tumor Xenografts in Nude Mice

BALB/c female nude mice (4–5 weeks old) used in this study were housed and maintained under standard NIH protocol, which were purchased from Beijing Vital River Laboratory Animal Technology (Beijing, China). MDA-MB-231 (1 × 10^7^) cells were inoculated into a nude mice breast pad. Approximately ten days later, when the average tumor volume reached 90–110 mm^3^, the mice were randomly divided into 4 groups–control group, ATO group, C20 group and ATO+C20 group. ATO (2 mg/kg mice body weight) and FEN1 inhibitor, C20 (10 mg/kg mice body weight) were administered intraperitoneally on alternate days. Tumor sizes were measured by a vernier caliper on alternate days, and tumor volumes (mm^3^) were calculated as length × width 2/2. Nine days later, all mice were euthanized. All of the animal experiments conformed to the Guide for Care and Use of Laboratory Animals and were approved by the Animal Care and Use Committee of China Medical University.

### Western Blot Assay

The harvested cells were solubilized in 1% Triton lysis buffer, and the protein concentration was determined using the Lowry method. After eluted by boiling water at 95°C for 5 min with 3x sampling buffer, the samples were separated using SDS-PAGE and electrophoretically transferred onto a PVDF membrane. Membranes were blocked by 5% skimmed milk in TBST [10 mM Tris (pH 7.4), 150 mM NaCl and 0.1% Tween-20] at room temperature for 2 h and incubated with FEN 1 (Abcam, CA, USA), PARP, γH2AX, p-p38, p38, p-JNK, JNK, Lamin A/C (Cell Signaling Technology, MA, USA), β-actin, BAX, BCL-2, Nrf2 (Santa Cruz Biotechnology, CA, USA) primary antibodies at 4°C overnight, followed by the incubation with monoclonal anti-rabbit or mouse secondary antibodies (Santa Cruz Biotechnology, CA, USA) for 30 min at room temperature. Blots were detected using an enhanced chemiluminescence reagent (SuperSignal Western Pico Chemiluminescent Substrate; Pierce Biotechnology, Rockford, IL, USA) and visualized using the Electrophoresis Gel Imaging Analysis System (DNR Bio-Imaging Systems, Jerusalem, Israel).

### Statistical Analysis

Differences between the two groups were analyzed using Student's *t*-test and are presented as the mean ± standard deviation. SPSS 17.0 computer software (SPSS, Inc., Chicago, IL, USA) was used for statistical analysis. *P* <0.05 was considered to indicate a statistically significant difference.

## Results

### FEN1 Inhibition Enhanced ATO-induced Cytotoxity and Growth Suppression

**Figure 1:**
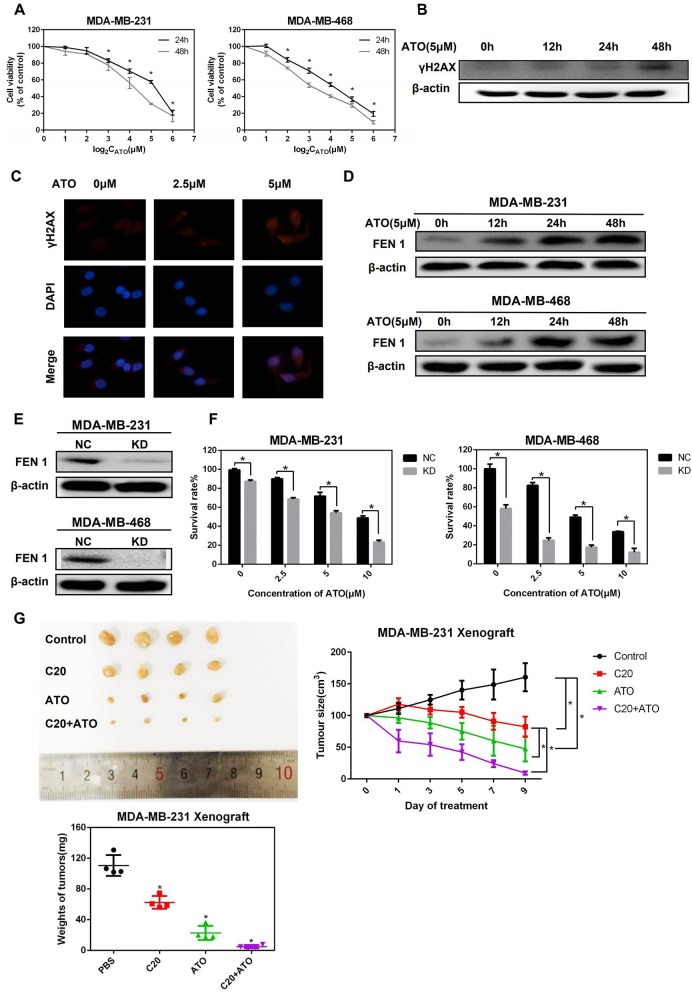
ATO upregulated FEN1 expression and FEN1 knockdown increased the sensitivity of TNBC cells to ATO. **(A)** MDA-MB-231 and MDA-MB-468 cells were treated with different concentrations (0, 1, 2, 4, 8, 16, 32 μM) of ATO for 24 and 48 h, respectively. Then the cell viability was detected by MTT assay. *P < 0.05, data are presented as mean ± standard deviation. **(B)** The MDA-MB-231 cells were treated with 5 mM ATO for the indicated times. Then the expression of γH2AX was detected by western blot. β-actin was used as an internal control. **(C)** The expression of γH2AX was detected by immunofluorescence. **(D)** The MDA-MB-231 and MDA-MB-468 cells were treated with ATO (5 μM) for the indicated time. Then the expression of FEN1 was detected by western blot. β-actin was used as an internal control. **(E)** The MDA-MB-231 and MDA-MB-468 cells were transfected with FEN1-specific siRNAs and the expression levels were measured by western blot. β-actin was used as an internal control. **(F)** Cells were transfected with FEN1-specific siRNA followed by treatment with different doses of ATO (0, 2.5, 5, and 10 μM) for the indicated time points. Then the cell viability was detected by MTT assay. *P < 0.05, data are presented as mean ± standard deviation. **(G)** Female nude mice were injected with MDA-MB-231 cells into a nude mice breast pad. Tumor-bearing mice were intraperitoneally injected with PBS, ATO (2 mg/kg), C20 (10 mg/kg), or combination (2 mg/kg ATO+10 mg/kg C20), and images of xenograft tumor were obtained from the different treatment groups. Analysis of weight and volume of subcutaneous tumors and measured to draw the growth curve of the tumor.

To determine the effect of ATO on TNBC cells, the cell viability of MDA-MB-231 and MDA-MB-468 cells with the treatment of ATO was evaluated by MTT. The results showed that ATO suppressed TNBC cells growth in a time- and dose- dependent manner (Figure 1A). The IC50s of ATO at 24 and 48 h for MDA-MB-231 cell were 19.88 ± 3.98 and 11.22 ± 2 μM, and that for MDA-MB-468 cells were 10.46 ± 0.92 and 6.02 ± 0.91 μM, respectively.

To further determine whether ATO could trigger DNA damage in TNBC cells, the expression of γH2AX, a DNA damage marker, was detected by western blot and immunofluorescence. It was shown that the expression of γH2AX was up-regulated after the treatment of ATO in MDA-MB-231 cells (Figures 1B,C). Next, to investigate whether FEN1 is involved in the repair process of DNA damage caused by ATO in TNBC cells, the change of FEN1 protein expression by the treatment of ATO was detected. As a result, the expression of FEN1 was increased at 12 h and kept at the high levels for 36 h in both of two TNBC cell lines (Figure 1D).

Then, the sensitivity of TNBC cells to ATO was detected after FEN1 was knocked down (Figure 1E). As shown in Figure 1F, the cell viability was significantly inhibited by the combination treatment of FEN1-KD and ATO, indicating that silencing FEN1 could increase the sensitivity of TNBC cells to ATO.

Furthermore, the effect of the combination of ATO and FEN1-KD was also examined *in vivo*, using a xenograft model in mice. For this study, a previously reported FEN1 inhibitor compound 20 (C20) was used (22, 23). C20 is an N-hydroxyl urea derivative that specifically inhibits FEN1 activity, which is the most potent FEN1 inhibitor tested at the time (24). The result showed that although the treatment of C20 or ATO alone suppressed the size and weight of tumors, the inhibitory effect of combination treatment was strongest (Figure 1G). All these results above indicated that the inhibition of FEN1 could enhance the anti-tumor effect of ATO, both *in vitro* and *in vivo*.

### Low Doses of ATO Inhibited TNBC Cell Proliferation Through ROS-induced Apoptosis

**Figure 2:**
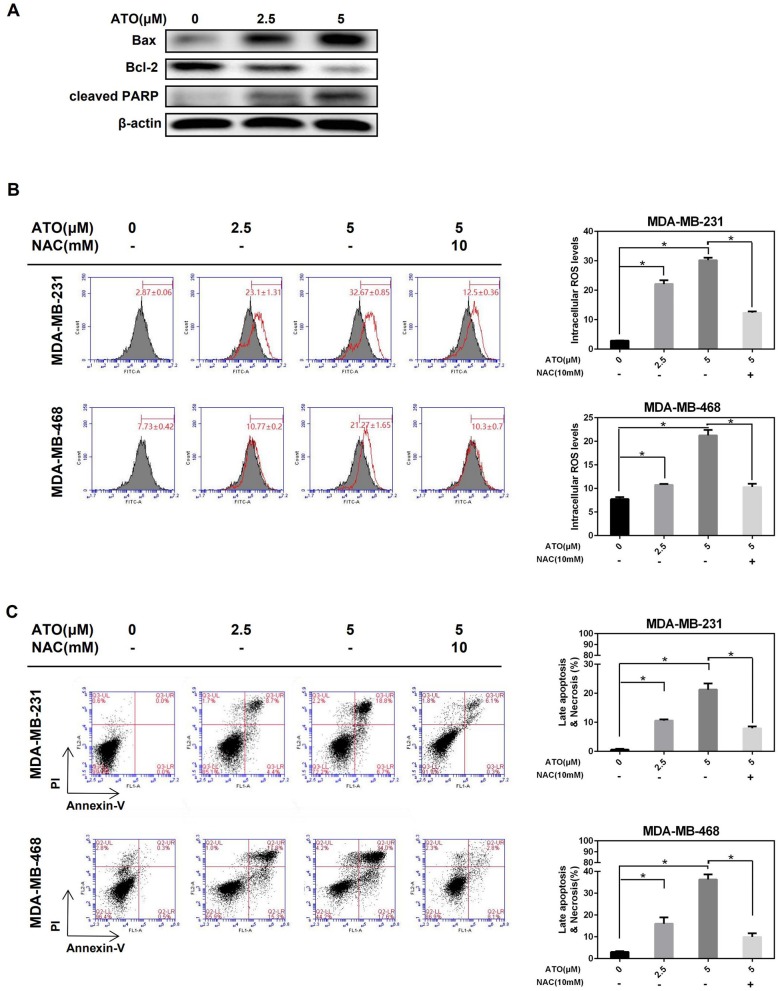
ATO induced apoptosis and necrosis by inducing ROS generation. **(A)** MDA-MB-231 cells were treated with various concentrations of ATO, then the expression level of Bax, Bcl-2, and PARP was detected by western blot. β-actin was used as an internal control. **(B,C)** The MDA-MB-231 and MDA-MB-468 cells were pretreated with or without 10 mM NAC for 1 h and the increasing concentrations (0, 2.5, and 5 μM) of ATO. **(B)** ROS were detected with carboxy-H2DCFDA. *P < 0.05. **(C)** Cell apoptosis and necrosis was evaluated by flow cytometry after annexin V and PI double staining. *P < 0.05.

Subsequently, a low dose of ATO (5 μM), which is below IC50, was selected to investigate whether ATO inhibited proliferation is related to apoptosis induction in TNBC cells. After treated with 0, 2.5, and 5 μM ATO for 48 h, the expression of apoptosis-related proteins was detected by western blot. As a result, the expression of Bax and cleaved-PARP was gradually increased, whereas the expression of Bcl-2 was decreased in MDA-MB-231 cells (Figure 2A), indicating ATO could induce apoptosis.

Then, whether ATO induced apoptosis is mediated by ROS was further investigated. Figure 2B showed that ATO significantly enhanced the generation of ROS, while this enhancement was obviously blocked by the pretreatment of inhibitor NAC in both MDA-MB-231 and MDA-MB-468 cell lines. Consistent with the trend of ROS change, the proportion of cell apoptosis, especially late apoptosis, was also induced by ATO-treatment alone, and reversed by NAC (Figure 2C). These results indicated that low doses of ATO could induce apoptosis by promoting ROS production in TNBC cells.

### Inhibition of FEN1 Increased ATO-induced ROS Accumulation and Consequent Cell Death

**Figure 3:**
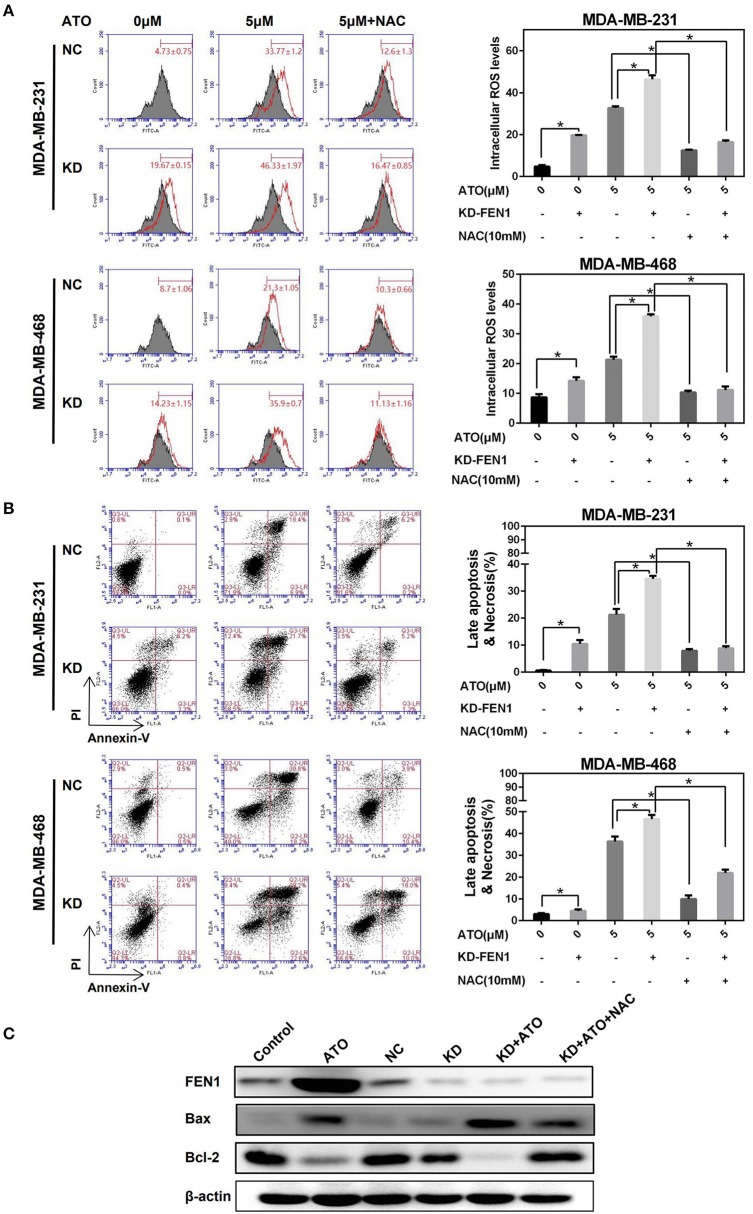
FEN1 knockdown increased ATO-induced ROS and cell death. **(A,B)** The MDA-MB-231 and MDA-MB-468 cells were transfected with FEN1-specific siRNA followed by treatment with or without NAC, then different doses of ATO (0 and 5 μM) for 48 h. **(A)** ROS were detected with carboxy-H2DCFDA. *P < 0.05. **(B)** Cell death was evaluated with annexin V and PI double staining by flow cytometry. *P < 0.05. **(C)** The MDA-MB-231 cells were transfected with FEN1-specific siRNA prior to pretreatment with or without NAC for 1 h and then ATO (5 μM). Cells incubated with serum-containing medium served as untreated controls. The expression of FEN1, Bax and Bcl-2 was measured by western blot. β-actin was used as an internal control.

To investigate whether FEN1 inhibition increased ATO sensitivity in TNBC is related to ROS generation, MDA-MB-231 and MDA-MB-468 cells were transfected with FEN1 siRNA or control siRNA, respectively, then treated with ATO for 48 h, with or without NAC 10 mM pretreatment. As shown in Figure 3A, comparing with the slight increase of ROS accumulation induced by FEN1-KD or ATO alone, the combination of FEN1-KD and ATO significantly enhanced ROS accumulation, which could be reversed by NAC in MDA-MB-231 and MDA-MB-468 cell lines.

Silencing FEN1 or using ATO alone led to mild apoptosis, but the combination of FEN1-KD and ATO, significantly induced apoptosis, which was also reversed by NAC (Figure 3B). Similar with the trend of ROS generation. Moreover, when detecting the ratio of Bax/Bcl-2 by western blot, it was showed that although FEN1-KD could not change the Bax/Bcl-2 ratio, this ratio was slightly increased by ATO-treatment, and further increased by the combination of FEN1-KD and ATO, which also could be reversed by NAC (Figure 3C).

### Inhibition of FEN1 Activates p38/JNK Pathway Induced by ATO

**Figure 4:**
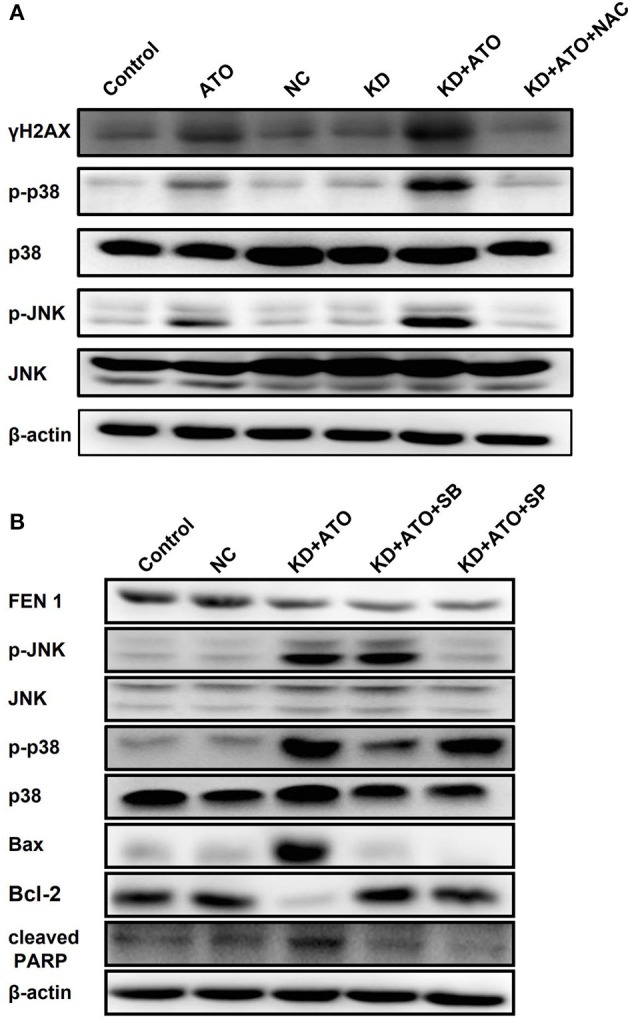
Knockdown of FEN1 increased ATO-induced DNA damage and activated p38/JNK pathway. **(A)** The MDA-MB-231 cells were transfected with FEN1-specific siRNA prior to pretreatment with or without NAC for 1 h and then the treatment of ATO (5 μM). Cells incubated with serum-containing medium served as untreated controls. The expression of γH2AX, p38, p-p38, JNK, and p-JNK was measured by western blot. β-actin was used as an internal control. **(B)** The MDA-MB-231 cells were treated with ATO (5 μM) followed by the transfection with FEN1-specific siRNAs, then pretreated with or without 10 mM SP600125 or 5 mM SB203580 for 1 h. After that, the expression of FEN1, p38, p-p38, JNK, p-JNK, Bax, Bcl-2, and PARP was measured by western blot. β-actin was used as an internal control.

It was reported that ATO could induce apoptosis via activating p38 and JNK pathway (25). Therefore, we suspected that FEN1 inhibition might enhance ATO-induced apoptosis by p38 and JNK pathway. Figure 4A showed that FEN1-KD had no significant effect on p38 and JNK, ATO alone could slightly activate p38 and JNK. Moreover, FEN1-KD combined with ATO significantly increased the phosphorylation level of p38 and JNK, which was reversed by NAC. These results indicated that the accumulation of ROS in TNBC could activate p38 and JNK pathway and induce cell death.

Furthermore, pretreatment with JNK and p38 inhibitors, SP600125 (Calbiochem, Germany) and SB203580 (Promega, WI, USA), blocked the activation of JNK and p38 signaling pathways activated by the combination of FEN1 knockdown and ATO (Figure 4B). The above results indicated that the combination of ATO and FEN1 inhibition could active p38 and JNK pathway by promoting ROS accumulation, finally leading to apoptosis in TNBC cells.

### Silencing FEN1 Reinforced the Inhibiting Effect of ATO by Inhibiting Nrf2 Nuclear Translocation and Decreasing GSH Level in TNBC Cells

**Figure 5:**
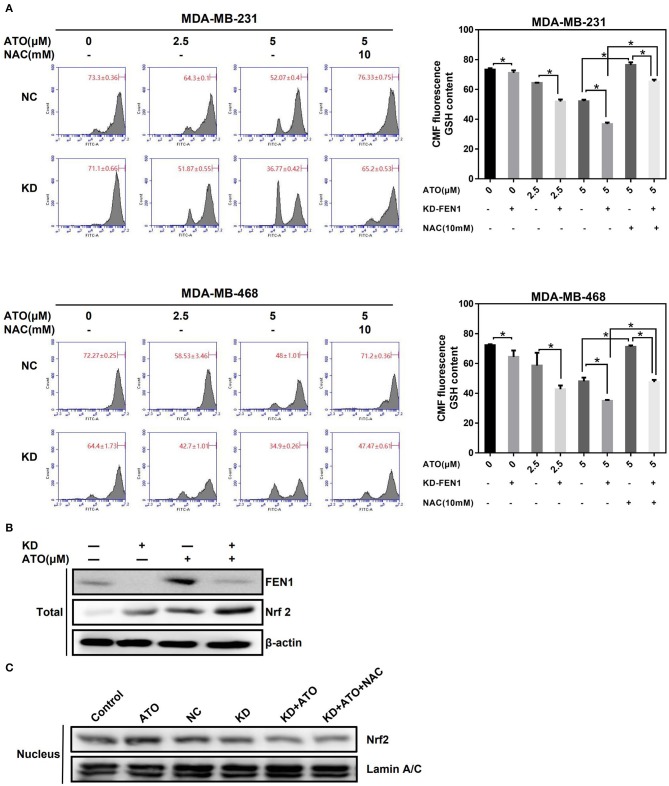
FEN1 knockdown in combination with ATO decreased GSH level and inhibited Nrf2 nuclear translocation. **(A)** The MDA-MB-231 and MDA-MB-468 cells were transfected with FEN1 specific siRNAs prior to the pretreatment with or without NAC for 1 h, and then giving increasing doses of ATO (0, 2.5, and 5 μM). GSH was evaluated using Green CMFDA staining by flow cytometry. *P < 0.05. **(B)** The MDA-MB-231 cells were transfected with FEN1-specific siRNAs prior to the treatment with or without ATO. The expression of FEN1 and Nrf2 was detected by western bolt. β-actin was used as an internal control. **(C)** The MDA-MB-231 cells were given with different treatment (Control, ATO 5 μM, NC-siRNA, FEN1-siRNA, FEN1-siRNA+ATO, FEN1-siRNA+ATO+NAC 10 mM), and the nucleoprotein was extracted for the detection of Nrf2 by western blot. Lamin A/C was used as an internal control.

Nrf2, key factor of antioxidant systems, was known to be able to protect cells against oxidative stress; improving nuclear translocation of Nrf2 can promote the synthesis and utilization of GSH, and in turn inhibit the proapoptotic effects of ATO on tumor cells (26, 27). To clarify the mechanism of FEN1-KD enhancing the effect of ATO on ROS accumulation. MDA-MB-231 and MDA-MB-468 cells were transfected with FEN1-siRNA or NC-siRNA, followed by ATO for 48 h, with or without NAC 10 mM pretreatment for 1 h, and the changes in GSH levels were detected by flow cytometry. The results showed that GSH levels were gradually decreased with the increasing concentration of ATO both in NC-siRNA and FEN1-KD cells (Figure 5A). Nevertheless, the levels of GSH were significantly lower in FEN1-KD cells than that in NC-siRNA cells. Furthermore, GSH levels were restored with NAC pretreatment in both of these cells, which was consistent with previous studies (28), GSH levels were restored after NAC pretreatment in both groups (Figure 5A).

**Figure 6:**
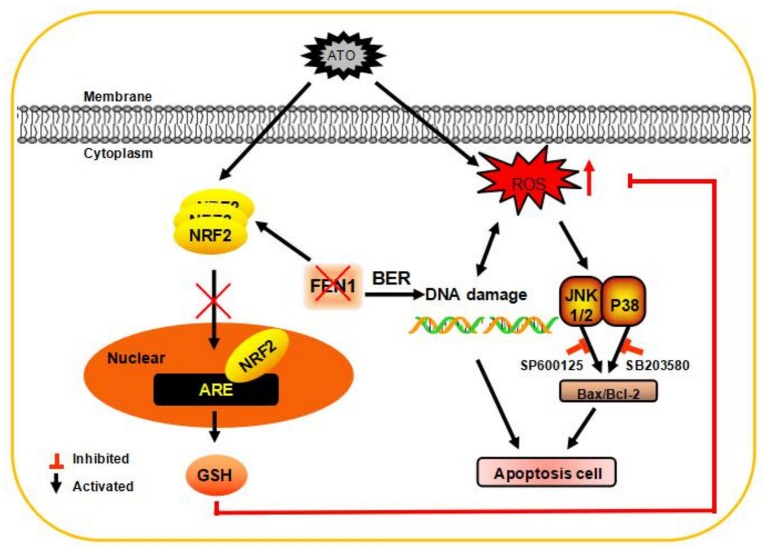
A schematic diagram of the application of ATO after inhibiting the expression of FEN1 to inhibit tumor growth in TNBC cells. FEN1 expression was increased after ATO treatment, and the inhibition of FEN1 resulted in slightly increase in ROS production. FEN1 knockdown in combination with ATO significantly reduced GSH level, and then increased intracellular ROS, through reducing Nrf2 nuclear transportation. Finally, the apoptosis and DNA damage were induced by ROS generation in MDA-MB-231 and MDA-MB-468 cells.

Next, we detected the expression and the nuclear localization of Nrf2 after different treatment. It was shown that the total protein expression of Nrf2 was increased after silencing FEN1 or ATO treatment alone and was further raised after combined treatment (Figure 5B). However, the nuclear translocation of Nrf2 in FEN1-KD and ATO combination-treated cells was decreased compared to ATO treated cells and FEN1-KD cells, which had the same trend with GSH. In addition, the levels of Nrf2 translocation could not be reverses by NAC (Figure 5C). These dates indicated that increasing GSH depletion and lower Nrf2 translocation reduces the production of GSH, inhibits the scavenging of ROS (Figure 6).

## Discussion

In physiological conditions, normal cells could maintain a lower ROS level than tumor cells and tolerate a certain degree of oxidative stress (29). However, these tumor cells have more peroxiredoxins to protect from oxidative damage, suggesting that tumor cells are more reliant on the antioxidant system and more sensitive to exogenous oxidative stimuli. The difference between normal cells and tumor cells can provide a biochemical basis for ROS-mediated selective anti-tumor therapy. In this study, we found that ATO could enhance ROS levels in TNBCs, while FEN1-KD could further increase the ROS accumulation. These results indicate that FEN1 has an important role in maintaining intracellular homeostasis to some extent of ROS and could partly protect cells from oxidative damage. Meanwhile, in colorectal cancer and tongue squamous cell carcinoma, DNA damage could lead to ROS accumulation, causing cell apoptosis (30, 31). Therefore, we speculate that ATO could induce DNA damage by promoting ROS accumulation and FEN1-KD lead to the obstruction of DNA repair. All of these can cause more DNA damage, which promote more ROS accumulation. As a result, further increasing ROS will cause more DNA damage, that positive feedback triggers cells apoptosis.

Many chemotherapeutic agents, such as Doxorubicin and Paclitaxel, can be used to treat TNBCs, which could promote ROS accumulation, in turn lead to cell death by causing DNA damage (32). Nevertheless, overexpression of DNA repair enzymes can repair DNA damage more efficient, which related to the mechanisms of drug-resistance. For example, overexpression of DNA polymerase B, a DNA repair enzyme in the BER pathway, is associated with etoposide resistance in small cell lung cancer (33). And using FEN1 inhibitors in lung and ovarian cancer could increase the antineoplastic effect of cisplatin and paclitaxel, by further enhancing the DNA damage (9, 13). However, those studies did not clarify whether the ROS-induced oxidative damage could be further enhanced after inhibition of FEN1. Our results showed that FEN1 inhibition could improve the anti-tumor effect of ROS inducer ATO by promoting ROS accumulation and reduce the application concentration of ATO. These results suggested that FEN1 can protect TNBCs from oxidative damage and providing references for combination usage of FEN1 inhibitor and ATO.

Although ROS inducers can effectively induce tumor cell apoptosis in many types of tumors, some recent studies have suggested that abnormally high ROS levels may cause drug resistance (34). Studies have shown that the tumor cells, which resist to paclitaxel, doxorubicin, or platinum drugs, have higher antioxidant capacity (34). Multidrug-resistant leukemia cells HL-60 with high expression of catalase protect from the cytotoxic effects of hydrogen peroxide (35). In ATO-resistant acute promyelocytic leukemia cells, resistance to ATO is associated with upregulation of heme oxygenase1 (HMOX1), superoxide dismutase 1 (SOD1), and GSH (26). Nrf2, a key protein that regulates many antioxidant enzymes, protects cells against oxidative stress. Previous studies have shown that improving nuclear translocation of Nrf2 can promote the synthesis and utilization of GSH, and in turn inhibit the proapoptotic effects of ATO on tumor cells (26). Inhibition of GSH biosynthesis can induce apoptosis in TNBC cells, and GSH can be a potential therapeutic target for TNBC (36). In this study, we found that using ATO after silencing FEN1 increases the total protein expression of Nrf2; nevertheless, its nuclear translocation decreases. Increasing GSH depletion and lower Nrf2 translocation reduces the production of GSH, inhibits the scavenging of ROS. The specific mechanism of the combination of FEN1 knockdown and ATO reduced the translocation of Nrf2 might be as follows. Activated Nrf2 is bound to the antioxidant transcription element (ARE) in the promoter region of phase 2 detoxification enzyme genes and antioxidant genes. It activates the transcription of these genes, so that cells can gain the enhanced resistance to oxidative damage or inflammation, avoiding the detrimental effects. In FEN1 gene promoter, there is an ARE-like sequence, which is a binding site to recruite Nrf2 (37). Therefore, it is reasonable to presume that FEN1 knockdown can reduce Nrf2 translocation as a feedback, and it needs further experiments to be clarified.

In conclusion, our results suggested that FEN1 can protect TNBCs from oxidative damage. Furthermore, inhibition of FEN1 expression could improve the anti-tumor effect of ROS inducer ATO. We believe that combination of FEN1 inhibitors and low doses of ATO could be a promising therapeutic approach for TNBC patients.

## Ethics Statement

The animal study was reviewed and approved by The animal study was approved by the Ethics Committee on Animal Care in China Medical University and all the experiments conform to the relevant regulatory standards.

## Author Contributions

Y-ET, X-FC, and TW conceived and designed the study. XX did the experiments and analyzed results, and written the manuscript. L-BG, LX, M-MD, K-ZH, SS, X-JQ, and Y-PL conducted experimental guidance. X-FC and Y-ET revised the manuscript.

### Conflict of Interest

The authors declare that the research was conducted in the absence of any commercial or financial relationships that could be construed as a potential conflict of interest.

## References

[1] Ferlay J Soerjomataram I Dikshit R Eser S Mathers C Rebelo M. Cancer incidence and mortality worldwide: sources, methods and major patterns in GLOBOCAN 2012. Int J Cancer. (2015) 136:E359–86. 10.1002/ijc.29210 25220842

[2] Trivers KF Lund MJ Porter PL Liff JM Flagg EW Coates RJ. The epidemiology of triple-negative breast cancer, including race. Cancer Causes Control. (2009) 20:1071–82. 10.1007/s10552-009-9331-1 19343511 PMC4852686

[3] Hammond ME Hayes DF Wolff AC Mangu PB Temin S. American society of clinical oncology/college of american pathologists guideline recommendations for immunohistochemical testing of estrogen and progesterone receptors in breast cancer. J Oncol Pract. (2010) 6:195–7. 10.1200/JOP.777003 21037871 PMC2900870

[4] Foulkes WD Smith IE Reis-Filho JS. Triple-negative breast cancer. N Engl J Med. (2010) 363:1938–48.393 10.1056/NEJMra1001389 21067385

[5] Costa R Shah AN Santa-Maria CA Cruz MR Mahalingam D Carneiro BA Targeting epidermal growth factor receptor in triple negative breast cancer: new discoveries and practical insights for drug 384 development. Cancer Treat Rev. (2017) 53:111–9. 10.1016/j.ctrv.2016.12.010 28104566

[6] Lan L Nakajima S Wei L Sun L Hsieh CL Sobol RW. Novel method for site-specific induction of oxidative DNA damage reveals differences in recruitment of repair proteins to heterochromatin and euchromatin. Nucleic Acids Res. (2014) 42:2330–45. 10.1093/nar/gkt1233 24293652 PMC3936713

[7] Lieber MR. The FEN-1 family of structure-specific nucleases in eukaryotic DNA replication, recombination and repair. Bioessays. (1997) 19:233–40. 10.1002/bies.950190309 9080773

[8] Shen B Singh P Liu R Qiu J Zheng L Finger LD. Multiple but dissectible functions of FEN-1 nucleases in nucleic acid processing, genome stability and diseases. Bioessays. (2005) 27:717–29. 10.1002/bies.20255 15954100

[9] He L Luo L Zhu H Yang H Zhang Y Wu H FEN1 promotes tumor progression and confers cisplatin resistance in non-small-cell lung cancer. Mol Oncol. (2017) 11:1302–3. 10.1002/1878-0261.12118 28861953 PMC5579339

[10] Lam JS Seligson DB Yu H Li A Eeva M Pantuck AJ Flap endonuclease 1 is overexpressed in 408 prostate cancer and is associated with a high Gleason score. BJU Int. (2006) 98:445–51. 10.1111/j.1464-410X.2006.06224.x 16879693

[11] Posadas EM Al-Ahmadie H Robinson VL Jagadeeswaran R Otto K Kasza KE. FYN is overexpressed in human prostate cancer. BJU Int. (2009) 103:171–7. 10.1111/j.1464-410X.2008.08009.x 18990162 PMC2741693

[12] Nikolova T Christmann M Kaina B. FEN1 is overexpressed in testis, lung and brain tumors. Anticancer Res. (2009) 29:2453–9. 19596913

[13] He L Yang H Zhou S Zhu H Mao H Ma Z. Synergistic antitumor effect of combined paclitaxel with FEN1 inhibitor in cervical cancer cells. DNA Repair. (2018) 63:1–9. 10.1016/j.dnarep.2018.01.003 29358095

[14] Singh P Yang M Dai H Yu D Huang Q Tan W. Overexpression and hypomethylation of flap endonuclease 1 gene in breast and other cancers. Mol Cancer Res. (2008) 6:1710–7. 10.1158/1541-7786.MCR-08-0269 19010819 PMC2948671

[15] Wang K Xie C Chen D. Flap endonuclease 1 is a promising candidate biomarker in gastric cancer and is involved in cell proliferation and apoptosis. Int J Mol Med. (2014) 33:1268–74. 10.3892/ijmm.2014.1682 24590400

[16] Zhou L Dai H Wu J Zhou M Yuan H Du J. Role of FEN1 S187 phosphorylation in counteracting oxygen-induced stress and regulating postnatal heart development. Faseb J. (2017) 31:132–47. 10.1096/fj.201600631r 27694478 PMC5161519

[17] Schumacker PT. Reactive oxygen species in cancer: a dance with the devil. Cancer Cell. (2015) 27:156–7. 10.1016/j.ccell.2015.01.007 25670075

[18] Chen H Gu S Dai H Li X Zhang Z. Dihydroartemisinin sensitizes human lung adenocarcinoma A549 cells to arsenic trioxide via apoptosis. Biol Trace Elem Res. (2017) 179:203–12. 10.1007/s12011-017-0975-5 28261759

[19] Elia AC Magara G Caruso C Masoero L Prearo M Arsieni P. A comparative study on subacute toxicity of arsenic trioxide and dimethylarsinic acid on antioxidant status in Crandell Rees feline kidney. (CRFK), human hepatocellular carcinoma. (PLC/PRF/5), and epithelioma papulosum cyprini. (EPC) cell lines. J Toxicol Environ Health A. (2018) 81:333–48. 10.1080/15287394.2018.1442758 29498595

[20] Mesbahi Y Zekri A Ghaffari SH Tabatabaie PS Admadian S Ghavamzadeh A. Blockade of JAK2/STAT3 intensifies the anti-tumor activity of arsenic trioxide in acute myeloid leukemia cells: novel synergistic mechanism via the mediation of reactive oxygen species. Eur J Pharmacol. (2018) 834:65–76. 10.1016/j.ejphar.2018.07.010 30012499

[21] Saha S Rashid K Sadhukhan P Agarwal N Sil PC. Attenuative role of mangiferin in oxidative stress-mediated liver dysfunction in arsenic-intoxicated murines. Biofactors. (2016) 42:515–32. 10.1002/biof.1276 27018134

[22] Exell JC Thompson MJ Finger LD Shaw SJ Debreczeni J Ward TA. Cellularly active N-hydroxyurea FEN1 inhibitors block substrate entry to the active site. Nat Chem Biol. (2016) 12:815–21. 10.1038/nchembio.2148 27526030 PMC5348030

[23] Tumey LN Bom D Huck B Gleason E Wang JM Silver D. The identification and optimization of a N-hydroxy urea series of flap endonuclease 1 inhibitors. Bioorg Med Chem Lett. (2005) 15:277–81. 10.1016/j.bmcl.2004.10.086 15603939

[24] He L Zhang Y Sun H Jiang F Yang H Wu H. Targeting DNA flap endonuclease 1 to impede breast cancer progression. EBioMed. (2016) 14:32–43. 10.1016/j.ebiom.2016.11.012 27852524 PMC5161424

[25] Alarifi S Ali D Alkahtani S Siddiqui MA Ali BA. Arsenic trioxide-mediated oxidative stress and genotoxicity in human hepatocellular carcinoma cells. Onco Targets Ther. (2013) 6:75–84. 10.2147/OTT.S38227 23404534 PMC3569381

[26] Nishimoto S Suzuki T Koike S Yuan B Takagi N Ogasawara Y. Nrf2 activation ameliorates cytotoxic effects of arsenic trioxide in acute promyelocytic leukemia cells through increased glutathione levels and arsenic efflux from cells. Toxicol Appl Pharmacol. (2016) 305:161–8. 10.1016/j.taap.2016.06.017 27317373

[27] Yamamoto T Suzuki T Kobayashi A Wakabayashi J Maher J Motohashi H. Physiological significance of reactive cysteine residues of Keap1 in determining Nrf2 activity. Mol Cell Biol. (2008) 28:2758–70. 10.1128/MCB.01704-07 18268004 PMC2293100

[28] Zheng CY Lam SK Li YY Ho JC. Arsenic trioxide-induced cytotoxicity in small cell lung cancer via altered redox homeostasis and mitochondrial integrity. Int J Oncol. (2015) 46:1067–78. 10.3892/ijo.2015.2826 25572414

[29] Hileman EO Liu J Albitar M Keating MJ Huang P. Intrinsic oxidative stress in cancer cells: a biochemical basis for therapeutic selectivity. Cancer Chemother Pharmacol. (2004) 53:209–19. 10.1007/s00280-003-0726-5 14610616

[30] Kutuk O Aytan N Karakas B Kurt AG Acikbas U Temel SG Biphasic ROS production, p53 and 40 BIK dictate the mode of cell death in response to DNA damage in colon cancer cells. PLoS ONE. (2017) 12:e0182809 10.1371/journal.pone.0182809 28796811 PMC5552129

[31] Shi X Wang L Li X Bai J Li J Li S. Dihydroartemisinin induces autophagy-dependent death in human tongue squamous cell carcinoma cells through DNA double-strand break-mediated oxidative stress. Oncotarget. (2017) 8:45981–93. 10.18632/oncotarget.17520 28526807 PMC5542242

[32] Yokoyama C Sueyoshi Y Ema M Mori Y Takaishi K Hisatomi H. Induction of oxidative stress by anticancer drugs in the presence and absence of cells. Oncol Lett. (2017) 14:6066–70. 10.3892/ol.2017.6931 29113247 PMC5661396

[33] Lawson MH Cummings NM Rassl DM Russell R Brenton JD Rintoul RC. Two novel determinants of etoposide resistance in small cell lung cancer. Cancer Res. (2011) 71:4877–87. 10.1158/0008-5472.CAN-11-0080 21642373 PMC3145147

[34] Ramanathan B Jan KY Chen CH Hour TC Yu HJ Pu YS. Resistance to paclitaxel is proportional to cellular total antioxidant capacity. Cancer Res. (2005) 65:8455–60. 10.1158/0008-5472.CAN-05-1162 16166325

[35] Suzuki T Spitz DR Gandhi P Lin HY Crawford DR. Mammalian resistance to oxidative stress: a comparative analysis. Gene Expr. (2002) 10:179–91. 10.3727/000000002783992442 12173744 PMC5977517

[36] Miran T Vogg ATJ Drude N Mottaghy FM Morgenroth A. Modulation of glutathione promotes apoptosis in triple-negative breast cancer cells. Faseb J. (2018) 32:2803–13. 10.1096/fj.201701157R 29301945

[37] Chen B Zhang Y Wang Y Rao J Jiang X Xu Z. Curcumin inhibits proliferation of breast cancer cells through Nrf2-mediated down-regulation of Fen1 expression. J Steroid Biochem Mol Biol. (2014) 143:11–8. 10.1016/j.jsbmb.2014.01.009 24486718

